# Spindle cell variant of medullary thyroid carcinoma: a clinicopathologic study of four cases

**DOI:** 10.1186/s13000-021-01152-w

**Published:** 2021-11-27

**Authors:** Yan Xia Wang, Shou Jing Yang

**Affiliations:** Department of Pathology, Xijing Hospital, 4th Military Medical University, No. 169 Chang Le Xi Road,, Xi’an, 710032 Shaanxi China

**Keywords:** Spindle cell, Thyroid carcinoma, Morphology, Immunophenotype

## Abstract

**Background:**

Medullary thyroid carcinoma (MTC) is a malignant tumor derived from C cells. It accounts for about 10% of all thyroid malignancies. More than 14 histological variants have been described. Among them, spindle cell variant is extremely rare.

**Case presentation:**

Here we describe 4 cases of spindle cell variant of MTC collected from 2012 to 2019. Ultrasound showed solid and hypoechoic nodules. Three patients underwent total thyroidectomy and regional lymph node dissection, and 1 patient underwent thyroid mass resection. Histologically, the tumors showed spindle shaped cells in bundles or interlaced arrangement, separated by hyalinised fibrous stroma that contained amyloid deposits. Immunohistochemistry showed that the tumor cells were positive for calcitonin, chromogranin A, synaptophysin, CD56, and TTF-1, but negative for other lineage-specific markers.

**Conclusions:**

We report 4 rare cases of spindle cell variant of MTC. Due to its rarity and special morphology, the diagnosis of spindle cell variant MTC relies on its morphology and immunohistochemical markers to avoid misdiagnosis.

## Background

Medullary thyroid carcinoma (MTC) is a malignant tumor with neuroendocrine characteristics, originated from parafollicular C cells secreting calcitonin. It accounts for 5 to 10% of all thyroid malignancies. Most patients are asymptomatic with lymph node metastasis in the early stage or distant metastasis in the later stage, and its mortality accounts for 13.4% of all thyroid malignancies. More than 14 histological subtypes of MTC have been described in current WHO classification. Spindle cell variant MTC is a rare variant, and only 5 cases have been reported to date [1–5]. It is easy to mistake for other lesions due to its rarity and spindle cell morphology. Here we report 4 cases of MTC with predominantly spindle cell pattern in clinical and pathological features to enhance understanding of this type of MTC.

## Materials and methods

### Case presentation

Four cases of spindle cell variant MTC were collected from 2012 to 2019, including 2 males and 2 females, aged 26 to 54 years old. Patients visited hospital with the accidental discovery of painless neck mass accompanied by gradual enlargement. On clinical examination, mass was diffuse and firm, moving with deglutition. Family history was unremarkable. Biochemical examination showed normal serum parathormone level. Thyroid function test was within normal range. Ultrasound showed solid and hypoechoic nodules of 2 ~ 6 cm in diameter. Two cases occurred in the left lobe of thyroid, and another 2 cases occurred in the right lobe of thyroid, including 1 case with gravelly calcification and 1 case with ipsilateral lymph node metastasis.

### Methods

The tissue samples were fixed with 10% neutral formalin and embedded in paraffin. Tissue sections of 4 ~ 5 μm thickness were prepared and stained with hematoxylin and eosin (H&E), and additional sections were stained with Congo red. Immunohistochemical staining was performed using Dako EnVision Peroxidase detection system on Roche Ventana Benchmark XT autostainer (Ventana Medical Systems, Tucson, AZ). The primary antibodies panel consisted of calcitonin (CT) (clone SP17, Ready-to-Use), chromogranin A (CgA) (SP12, Ready-to-Use), synaptophysin (Syn) (SY38, 1:100), CD56 (1B6, 1:50, Novocastra, Newcastle upon Tyne, UK), TTF-1 (SPT24, prediluted), Thyroglobulin (TG) (2H11 + 6E1), Ki-67 (MIB-1, 1:100), carcinoembryonic antigen (CEA) (12–140-10, Ready-to-Use), cytokeratin (AE1/AE3, Ready-to-Use), epithelial membrane antigen (EMA) (E29, 1:100), vimentin (V9, 1:100), desmin (D33, 1:160), alpha-smooth muscle actin (SMA) (lA4, 1:100, Dako), HMB45 (HMB-45, 1:50), S-100 protein (polyclonal,1:200), HBME-1 (HBME-1, Ready-to-Use), galectin-3 (9C4, Ready-to-Use), CK19 (A53-B/A2.26, Ready-to-Use), and CD99 (12E7, 1:50). Unless otherwise stated, all antibodies were mouse monoclonal and from DAKO Corporation (Dako North America, Inc., Carpinteria, CA, USA). Appropriate positive and negative controls were run in parallel.

## Results

### Pathological morphology

The clinicopathological features of patients are summarized in Table 1. Grossly, the mass of the four patients had clear boundary, 2 ~ 6 cm in diameter, solid shape, soft texture and grayish-white or grayish-brown in color. Among them, the capsule of the tumor was intact in 3 patients, and incomplete in 1 patient.

**Table 1:** Clinicopathological variables of patients with spindle cell variant of medullary thyroid carcinoma

Case	Sex	Age	Tumor site	Tumor size (cm)	Histological type	Immunophenotype	Recurrence	Follow-up (months)	Status
1	F	54	LLT	2 × 2 × 2.5	spindle cell variant MTC	CT+, CgA +, Syn+, CD56+, TTF-1+, TG-, AE1/AE3+, Ki-67+ (5%)	_	60	Alive
2	M	26	RLT	6 × 3× 3	spindle cell variant MTC	CT+, CgA +, Syn+, CD56+, TTF-1+, TG-, CEA+, AE1/AE3+, SMA-, S-100-,HMB45-, CD99-, Ki-67+ (5%)	_	7	Alive
3	F	49	LLT	4 × 2× 2	spindle cell variant MTC	CT+, CgA +, Syn+, CD56+, TTF-1+, TG-, CEA+, vimentin+, SMA-, S-100-, HBME-1-, galectin-3-, CK19-, Ki-67+ (3%)	_	84	Alive
4	M	51	RLT	2.4 × 1.6 × 2.2	spindle cell variant MTC	CT+, CgA +, Syn+, CD56+,TTF-1+, TG-, CEA+ EMA+,vimentin+, SMA-, S-100-, HBME-1-, Ki-67+ (10%)	_	72	Alive

Abbreviations: F female, M male, LLT the left lobe of thyroid, RLT the right lobe of thyroid, MTC Medullary thyroid carcinoma

**Fig. 1:**
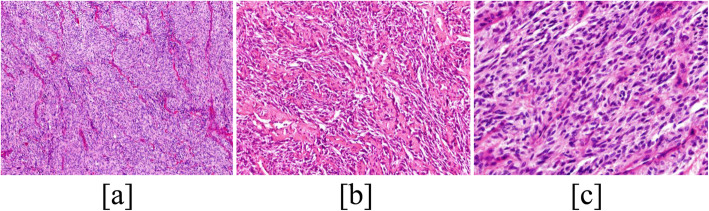
Histiological findings. Tumor show predominantly monomorphic spindle cells, arranged in fascicles or interweave pattern (**a**), separated by fibrovascular amyloid stroma (**b**) (H&E, original magnification, × 200). Tumor cells show short or long spindle nuclei, abundant cytoplasm and rare mitoses (**c**) (H&E, original magnification, × 400)

Histological examination of the specimens showed that the tumors consisted predominantly of monomorphic spindle cells, arranged in fascicles or interweaves (Fig. 1a), separated by fibrovascular or amyloid stroma (Fig .1b). Tumor cells showed short or long spindle nuclei, abundant cytoplasm, inconspicuous nucleoli and rare mitoses (Fig. 1c). The boundary between tumor tissue and peripheral thyroid tissue was clear.

### Congo red amyloid stain

Tumor tissue were positive (3/3) for Congo red staining.

### Immunohistochemistry

**Fig. 2:**
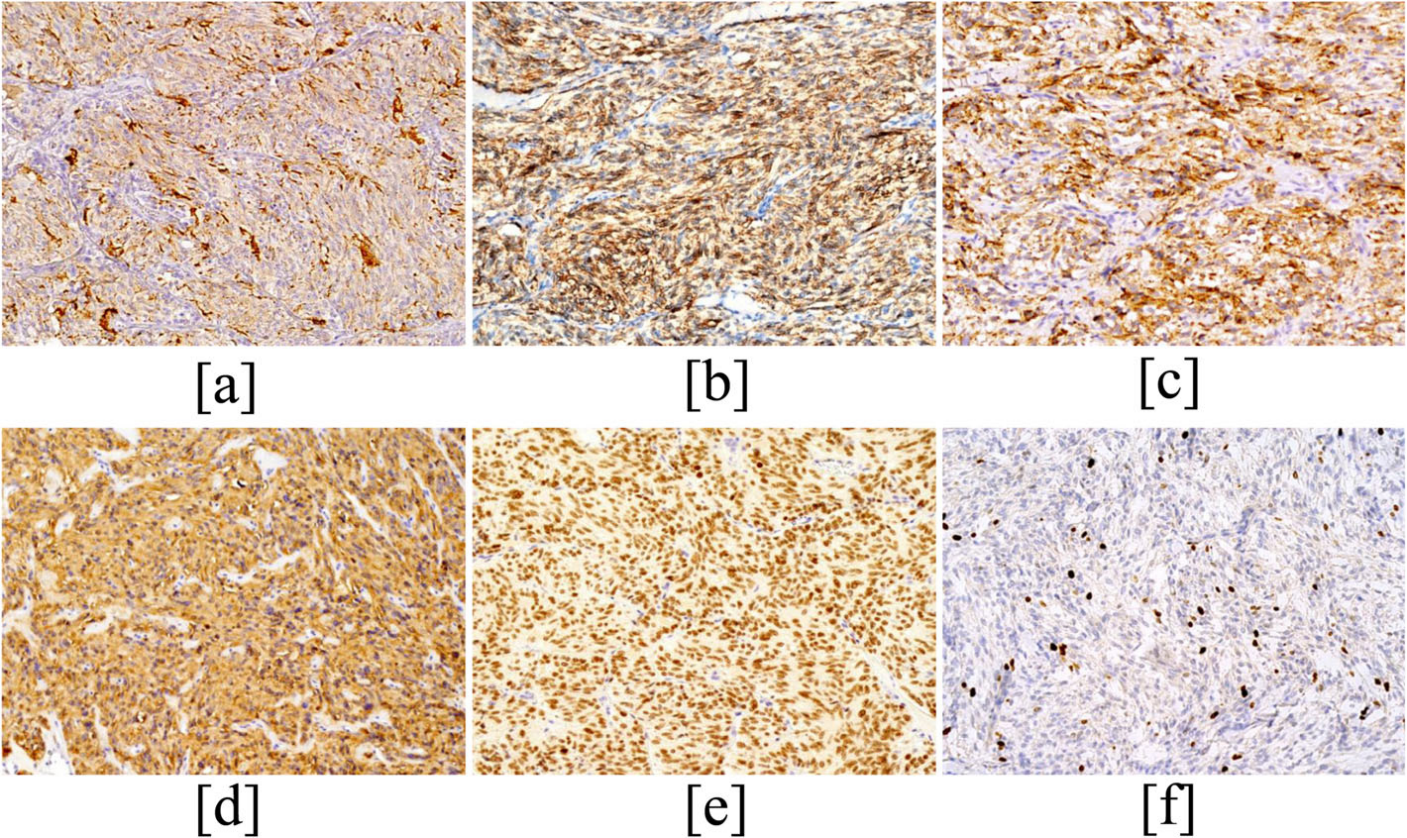
Immunohistochemistry. The tumor cells are positive for CT (**a**), CD56 (**b**), CgA (**c**) and Syn (**d**) in the cytoplasm (Original magnification, × 200). The tumor cells are positive for TTF-1(**e**) and Ki-67 (**f**) in the the nucleus (Original magnification, × 200)

The immunohistochemical results are summarized in Table 1. The tumor cells were positive for CT (Fig. 2a), CgA (Fig. 2b), Syn (Fig. 2c), CD56 (Fig. 2d), and TTF-1 (Fig. 2e) in all 4 cases. In addition, the tumor cells were also positive for AE1/AE3 (2/2), CEA (3/3), EMA (1/1) and vimentin (2/2). Other markers such as TG, Desmin, SMA, S-100, galectin-3, CK19, HBME-1, and CD99 were negative. The Ki-67 proliferative index of these tumors ranged approximately 3% ~ 10% (Fig. 2f).

### Treatment and follow-up

Three patients underwent total thyroidectomy and regional lymph node dissection, and 1 patient underwent thyroid mass resection. After a follow up of 7 ~ 84 months, all four patients were alive.

## Discussion

Spindle cell variant MTC is a rare type of MTC, and only 5 cases have been reported in literatures thus far [1–5]. We reviewed 99 cases of MTC confirmed in our hospital from 2012 to 2019, only 4 cases spindle cell variant MTC meet the criteria, accounting for 4% of all MTC.

MTC is a neuroendocrine tumor, accounting for 5% ~ 10% of thyroid malignant tumors [6]. The clinical symptoms of spindle cell variant MTC are consistent with MTC. MTC patients generally have no specific clinical symptoms, only presenting with a painless thyroid mass, sometimes presenting with intractable diarrhea, facial flushing or other neuroendocrine symptoms. In addition, serum calcitonin (sCT) is the most sensitive and specific marker for preoperative diagnosis of MTC [7], and serum CEA is synchronous with sCT in some MTC patients [8, 9], which is of reference value for diagnosis and prognosis of the disease. When the tumor causes compression or invasion to the surrounding tissues, the corresponding clinical symptoms may appear, and lymph node metastasis is easy to occur in the early stage. All the patients in our series presented with cervical mass without increase of sCT and CEA.

Histological diagnosis of MTC depends on its cytological feature, growth pattern, and amyloid deposition, as well as its immunophenotype. MTC with predominant spindle cell morphology is seen less commonly. The spindle cell variant MTC consists mainly of irregularly arranged bundles or interlaced spindle cells in which the stroma is separated by fibrous stroma or amyloid. Amyloid deposition is not a necessary condition for MTC diagnosis, since amyloid may not be present in atypical cases. The tumor cells showed short or long spindle nuclei, abundant cytoplasm, inconspicuous nucleoli and rare mitoses, as well as clear boundaries with surrounding tissues. Immunohistochemically, the most sensitive markers for the neoplasm are CT and calcitonin gene-related peptide, although they are not specific because they also can be found in endocrine tumors of nonthyroid origin, such as islet cell tumors and intestinal neuroendocrine carcinomas. In addition, MTC cells also are immunoreactive for cytokeratins, neuron-specific enolase, Syn, CEA, and CgA, whereas they are not stained by TG. In our cases, the tumors showed spindle cell morphology, the positive expressions of TTF-1, CT, CgA, Syn and CD56 and amyloid deposition, confirming the diagnosis as spindle cell variant MTC, which were consistent with those reported in the literature [10].

Spindle cell variant MTC is necessary to differentiate it from other primary or secondary thyroid tumors that show spindle cell morphology [11]. Other spindle cell tumors occurring in the thyroid gland, such as spindle cell atypical thyroid adenoma, spindle epithelial tumor with thymus-like differentiation, poorly differentiated thyroid carcinoma, and anaplastic thyroid carcinoma, may show spindle cell morphology and positive expression of epithelial cell markers, TTF-1 and TG, but they are negative for neuroendocrine markers. Therefore, it is easy to distinguish these tumors by comprehensive staining of CT, TTF-1, CgA, Syn and CD56. Also, spindle cell variant MTC can mimic some spindle cell mesenchymal tumors occurring in the thyroid gland, such as leiomyoma, peripheral schwannoma, and spindle cell melanoma. These tumors have specific markers, but are negative for neuroendocrine markers. Therefore, the morphology in conjunction with immunohistochemistry is sufficient to render a diagnosis of these tumors.

Surgical treatment is still the best treatment for spindle cell variant MTC. When surgical treatment is difficult to control, the efficacy of radioactive iodine treatment, in vitro irradiation or chemotherapy is poor. Therefore, early detection and treatment is the most effective way to improve the therapeutic effect. In our series, all patients received local resection or regional lymph node dissection. During follow-up of 7 ~ 84 months in our series, all patients were alive and had no recurrence.

In conclusion, spindle cell variant MTC is an extremely rare histological subtype. It is easy to diagnose and differentiate from other spindle cell lesions of thyroid by histological morphology, immunohistochemical staining with CT, CgA, Syn, CD56, and TTF-1.

## Data Availability

The datasets used and/or analyzed during the current study are available from the corresponding author upon reasonable request.

## Abbreviations

MTC: Medullary thyroid carcinoma
WHO: World Health Organization
CT: calcitonin
CgA: chromogranin A
Syn: synaptophysin
TG: Thyroglobulin
CEA: carcinoembryonic antigen
EMA: epithelial membrane antigen
